# Dissemination of evidence-based homeopathy before Brazilian medical institutions as a strategy to strengthen the homeopathic medical specialty

**DOI:** 10.1016/j.clinsp.2026.101127

**Published:** 2026-09-07

**Authors:** Marcus Zulian Teixeira

**Affiliations:** Department of Psychiatry, Faculdade de Medicina, Universidade de São Paulo (FMUSP), São Paulo, SP, Brazil

## Abstract

• Despite being part of the list of Brazilian medical specialties since 1980, homeopathy is not taught in most medical schools.
• This gap in medical students’ education about homeopathy is reflected in the lack of information and ignorance of future doctors.
• The application of unorthodox principles by homeopathy contributes to the spread of prejudices ingrained in medical culture.
• The text aims to clarify for the medical class the assumptions and scientific evidence of homeopathy.
• Dissemination of evidence-based homeopathy among Brazilian medical institutions contributes to the strengthening of the homeopathic medical specialty.

## Introduction

Homeopathy is a therapeutic model used worldwide that, in the last few decades, along with other approaches of integrative medicine, has been awakening growing interest among users, medical students, and doctors.^1^, ^2^, ^3^ Homeopathy provides an efficient and safe medical practice, aiming to understand and treat the patient-disease binomial according to an anthropological vitalist, globalizing, and humanistic approach,^4^^,^^5^ valuing the various aspects of the sick individuality.

Established by the German physician Samuel Hahnemann in 1796, homeopathy has been a recognized medical specialty by the Brazilian Federal Council of Medicine (CFM) since 1980 (Resolution CFM n° 1000/1980), with the title of specialist awarded by the Brazilian Medical Association (AMB) since 1990 (Resolution CFM n° 2,068/2013).^1^, ^2^, ^3^

Developing its activities in parallel with mainstream medicine, homeopathy disseminates its theoretical, practical, and scientific rationality in postgraduate courses of a broad sense, taught by training entities linked to the Brazilian Homeopathic Medical Association (AMHB). In 2004, following the CFM Resolution n° 1634/2002, it began to be offered in the medical residency program of the Federal University of the State of Rio de Janeiro (UNIRIO), Gaffrée and Guinle University Hospital. Currently, three more medical residency programs offer homeopathy as an option for service training: Regional Public Hospital of Betim (MG), Federal University of Mato Grosso do Sul (UFMS), and Cesmac University Center (AL).^1^, ^2^, ^3^

With consultations and procedures reimbursed by health insurance plans since 1985, homeopathy began to be provided in the public health services of the Brazilian Unified Health System (SUS), with thousands of specialist doctors practicing in the country. Despite the growing demand from the population for the therapy, a small number of Brazilian municipalities provide homeopathy within the SUS. In the last decades, initiatives in global medical education have made it possible to teach homeopathic principles in medical schools, incorporating mandatory and elective disciplines into basic curriculum,^1^, ^2^, ^3^ allowing theoretical information supported by scientific evidence and practical experiences to dissolve the prejudice and misinformation ingrained in the medical culture.^6^^,^^7^

Despite existing for over two centuries as a therapeutic option in various countries, homeopathy remains marginalized within the modern scientific rationality because it is based on unorthodox concepts that challenge dominant biomedical thinking. The homeopathic epistemological model employs the principle of cure by similitude (similarity), administering infinitesimal doses of unique and individualized medicines that, having been previously tested on healthy individuals, caused symptoms similar to those of sick individuals. To become a homeopathic medicine, the substance must undergo pathogenetic experimentation protocols in healthy human beings and have its primary effects described in the Homeopathic Materia Medica. Aiming to restore homeostatic balance, the art of homeopathic healing must be able to identify individual morbid susceptibilities, recognized through the totality of signs and symptoms manifested by the patient, in order to choose a medicine that has elicited a similar set of manifestations when tested on healthy experimenters.^8^^,^^9^

In view of homeopathy valuing psychic and emotional symptoms as aspects of high hierarchy in the set of human manifestations, whether in homeopathic pathogenetic experimentation or in understanding the etiopathogenesis of health disorders, this class of subjective characteristics is part of the homeopathic cure ideal. Medicines that suppress undesirable clinical manifestations without providing proportional psychic and emotional improvements do not satisfy the globalizing conception of the homeopathic curative process. Therefore, every individualized and well-conducted homeopathic treatment must act, in an integrated manner, on both psychic and emotional disorders as well as general and physical disorders, aiming to provide a state of physical, mental, social, and spiritual well-being.^8^^,^^9^

In short, the homeopathic model for treating diseases is based on four pillars, principles, or assumptions: principle of therapeutic similitude, homeopathic pathogenetic trial or experimentation, individualized medicine (therapeutic individualization), and dynamized or potentiated medicine (infinitesimal doses or ultra-dilutions). As we will cite below, these homeopathic assumptions are based on several lines of contemporary research, contrary to the misinformation commonly propagated.^8^^,^^9^

In this text, we will describe recent initiatives to discuss and disseminate evidence-based homeopathy before Brazilian medical institutions as a strategy to strengthen the homeopathic medical specialty.

### Initiatives to disseminate evidence-based homeopathy before Brazilian medical institutions

In the last decade, we have been working to spread homeopathic scientific evidence among Brazilian medical institutions, aiming to strengthen the homeopathic medical specialty in the face of the various attacks suffered from groups of pseudoskeptics supported by the mass media.

### Special Dossier: "Scientific Evidence for Homeopathy" (CREMESP, 2017)

With the aim of presenting an overview of homeopathic research to the medical community and society in general, the Technical Chamber of Homeopathy of the Regional Council of Medicine of the State of São Paulo elaborated the *Special Dossier: Scientific Evidence for Homeopathy* (CREMESP, 2017), which was published in three languages (Portuguese, English and Spanish) and made available in independent editions of open-access homeopathic scientific journals.^10^, ^11^, ^12^

Comprising nine narrative reviews of research in various fields of medical science (historical, social and political, medical education, pharmacological, basic, clinical, patient safety, and pathogenetic experimentation), this dossier includes hundreds of scientific articles that, by describing controlled experimental and clinical studies, highlight the “state of the art” of research in homeopathy.^10^, ^11^, ^12^

Despite the difficulties and limitations in conducting research in homeopathy, both due to methodological aspects and the lack of institutional and financial support, the body of experimental and clinical studies described in this dossier, which support homeopathic principles and confirm the efficacy and safety of the therapy in various health disorders, is a proof that “there is scientific evidence for homeopathy”, contrary to the prejudice falsely disseminated by pseudoskeptics and pseudoscientists.^13^

### “Homeopathy is not Placebo Effect”: Proof of Scientific Evidence for Homeopathy (AMB, 2023-2024)

After a series of attacks on the homeopathic medical specialty,^14^ the authors published in 2023 the electronic book (e-book, PDF format) in Portuguese, “*Homeopatia não é efeito placebo*”: *comprovação das evidências científicas da homeopatia,* with the aim of expanding and updating evidence-based homeopathy covered in the aforementioned *Special Dossier: Scientific Evidence for Homeopathy* (CREMESP, 2017),^10^, ^11^, ^12^ counting on the support of the Brazilian Medical Association (AMB). In 2024, the e-book was translated into English and Spanish (“*Homeopathy is not placebo effect”: proof of scientific evidence for homeopathy* / “*La homeopatía no és efecto placebo*”: *comprobación de las evidencias científicas en homeopatía*), and made available in open access editions at various platforms.^15^, ^16^, ^17^

Expanding knowledge in the field across thirteen interactive chapters, the e-book covers topics ranging from “homeopathic clinical epidemiology” to “pseudoskeptical and pseudoscientific strategies used in attacks on homeopathy”, including “pharmacological basis of the principle of similarity”, “experimental studies in biological models”, “randomized controlled clinical trials”, “systematic reviews, meta-analyses, and global reports,” and “observational studies,” among others.^15^, ^16^, ^17^

### I Webinar of Homeopathy of the Brazilian Federal Council of Medicine (CFM, 2025)

After being established in early 2025, the Technical Chamber of Homeopathy of the Brazilian Federal Council of Medicine held the 1^st^ Homeopathy Webinar on November of the same year (CFM, 2025), with the objective of “discussing in a free, independent, and well-founded manner the existing research in homeopathy in Brazil and in the world”.

The event, broadcast and made available online,^18^ “sought to offer a space for analysis and critical reflection on the available scientific production, evaluating the consistency and limitations of the evidence, as well as the challenges for teaching and research in this area of medical knowledge”.

The webinar program covered various areas of scientific research in homeopathy, namely: “Overview of research and experimental evidence in homeopathy: *in vitro,* laboratory, and clinical scientific studies”, “Critical evaluation of homeopathy as a medical therapy: what systematic reviews and meta-analyses tell us”, “Qualitative research in homeopathy: socio-historical-anthropological and quality of life studies” and “Teaching and research of homeopathy in university institutions in Brazil: the experience of the Federal University of the State of Rio de Janeiro (UNIRIO)”, among others.^18^

Similar to other activities promoting evidence-based homeopathy developed in the aforementioned Brazilian medical institutions, this online seminar highlighted the importance of discussing the clinical and scientific evidence of this specialty ‒ recognized by the CFM for 45-years ‒ and reinforced its role in humanized medical practice, characterized by listening, attention, and qualified care.

## Discussion

Beyond the implications for medical education and professional practice that an overview of homeopathic research might offer, the text also proposes a reflection on the challenges involved in generating and disseminating scientific evidence regarding a non-mainstream medical practice within the context of institutional or conventional medicine (biomedical model).

By employing unconventional assumptions ‒ specifically the application of the principle of therapeutic similarity through the administration of individualized medicines in infinitesimal doses ‒ homeopathy runs counter to logical-deductive reasoning (which proceeds from general rules and established truths to explain or predict a particular case), requiring that the physician and researcher adopt a counter-intuitive stance to accept the logic of homeopathy, which challenges common sense and conventional expectations.

Scientific research in homeopathy seeks to provide a foundation for these non-orthodox principles, which were broadly addressed in the works cited earlier (dossier, e-book and webinar). We will briefly discuss below some existing lines of research that support homeopathic pillars, with the aim of encouraging colleagues and researchers to delve deeper into the reference material.

### Basis of the principle of similitude in modern pharmacology

Based on the study of the pharmacological properties of dozens of medicinal substances of his time, in which he observed an opposite secondary reaction (indirect effect) of the organism after the primary action (direct effect) of drugs from various classes, Samuel Hahnemann proposed using substances that cause a set of signs and symptoms similar to the patient’s disorder with the aim of triggering a curative reaction (indirect effect) in the sick organism.

Cited since Hippocrates, the principle of similarity (vital or homeostatic reaction) finds its scientific basis on the “rebound effect” of modern drugs (indirect effect or paradoxical reaction of the organism), being described after the suspension or alteration of doses of numerous classes of medicines that act in a contrary or palliative way (principle of contrary) to the symptoms of diseases, aggravating the symptoms initially suppressed. The rebound effect is confirmed in hundreds of studies in clinical and experimental pharmacology.^19^^,^^20^

Expanding on this source of evidence, this line of research also describes the use of modern drugs according to the principle of therapeutic similarity (“new homeopathic medicines” proposal), employing the rebound effect (paradoxical reaction of the organism) in a curative way.^20^^,^^21^

### Clinical effectiveness of the individualized homeopathic medicine

According to Hahnemann, appropriate homeopathic treatment should prioritize the individualization of the single medicine according to the totality of the most peculiar and characteristic signs and symptoms of each patient in their various constitutional aspects (mental, general and physical), allowing that, for the same disease, each individual may receive distinct single medicines, according to their own physical, psychic, emotional, dietary, climatic, and other susceptibilities. This is a *sine qua non* condition of the homeopathic clinical epidemiology (homeopathic episteme).

Several Randomized Controlled Trials (RCTs) that disregarded the protocol of individualized medicine by administering the same medicine to several individuals with the same disease (exemplified by the indiscriminate use of *Arnica montana* for inflammatory processes),^22^ did not show significant results compared to placebo, as they violated the scientific rationale of the homeopathic model.^8^, ^9^ The same occurred with meta-analyses and systematic reviews that grouped RCTs with non-individualized medicines,^23^ unlike those that prioritized individualized therapy.^24^

In this context, the meta-analysis by Shang et al. (The Lancet, 2005)^25^ and other reports are included, which are discussed in detail in the chapter “Systematic reviews and global reports with negative results of homeopathy compared to placebo (Methodological flaws)” of the previously mentioned e-book (AMB, 2023‒2024).^15^, ^16^, ^17^

Besides this important epistemological bias of Shang et al.’ meta-analysis,^25^ which is often cited as indisputable proof that “the clinical effects of homeopathy are placebo effects”, other serious methodological flaws have been described in post-hoc analyses, such as: in the *first and main analysis* with all studies included (110 homeopathic RCTs versus 110 conventional RCTs, matched for disease and outcome), main outcome of the meta-analysis indicated in the original objective, both homeopathy and conventional medicine were significantly more effective than placebo; in the *second analysis,* including only studies with the largest number of participants (8 homeopathic RCTs and 6 conventional RCTs, not matched for disease and outcome), just conventional medicine were significantly more effective than placebo.^26^

Based only on this second and biased analysis (disregarding the first and main analysis with all 220 RCTs), the authors concluded that “there was weak evidence for a specific effect of homeopathic remedies, but strong evidence for specific effects of conventional interventions”, inferring that “the clinical effects of homeopathy are placebo effects”. This biased conclusion led the journal to proclaim in its editorial “The end of homeopathy”.^26^

Confirming that the clinical effects of homeopathy are not just placebo effects, a recent systematic review of all global systematic reviews of RCTs with meta-analyses was published in 2023,^27^ demonstrating that “global systematic reviews of homeopathic RCTs with meta-analyses reveal significant positive effects of homeopathy compared to placebo” and that “there was no support for the alternative hypothesis of no outcome difference between homeopathy and placebo”.

### Models of action of homeopathic infinitesimal doses (ultra-dilutions)

With the initial goal of avoiding intoxications and symptomatic aggravations that the principle of therapeutic similarity could cause in patients, Hahnemann proposed a pharmacotechinal method to the preparation of homeopathic medicines (dynamization or potentiation), in which substances are successively diluted and agitated in order to reduce the primary pathogenic effect. Later, he observed that these infinitesimal and imponderable preparations mobilized biological activity in various spheres of individuality.

Contrary to the orthodox dose-dependent pharmacological model (pharmacological plausibility), it is surprising to biomedical reasoning that substances in infinitesimal or ultra-diluted doses (dynamized or potentiated), in concentrations lower than Avogadro’s constant (6.02×10^23^ moL^-1^), may elicit some response in biological systems or living beings; this is the main target of criticism of the homeopathic model.

The ability of this medicinal “information” (contained in the infinitesimal doses of homeopathic ultra-diluted substances) to promote changes in organic systems in a manner analogous to ponderable doses has been studied in hundreds of experimental studies employing physicochemical and biological (*in vitro*, plants, and animals) research models, which are discussed in detail in the corresponding chapter of the previously mentioned e-book (AMB, 2023‒2024).^15^, ^16^, ^17^

In recent decades, physicochemical research models have been tested in several experimental methods (nuclear magnetic resonance, optical and Raman spectroscopy, electrical impedance, analytical methods, microscopic tools, photoluminescence, calorimetry, chromatography and electrical impedance, among others) and some hypotheses suggest scientific explanations for the phenomenon of the transmission of “information” about the primary effects of substances in ultra-diluted doses. Among them, we can cite research that studies the electromagnetic modifications of water according to the quantum electrodynamics, in which the aqueous solution would not represent an inert cluster of molecules but rather a dynamic medium, capable of selecting and catalyzing molecular reactions according to the various electromagnetic fields that occur within it.^28^, ^29^, ^30^

Through mathematical and experimental models, they infer that the electromagnetic field of a solute can generate certain stable coherence domains in the solvent (with specific structures and vibrations), producing “clusters” of water molecules (with specific sizes, shapes, and properties), like an electromagnetic signature of the solute in the water (“water memory”). Therefore, the organization of water would be a coherent, reproducible process associated with long-range, very low-intensity electromagnetic interactions, transmitting the ‘electromagnetic information of the solute’ initially diluted and succussed by the dynamization process.^28^, ^29^, ^30^

Regarding the biological research models (*in vitro,* plants, and animals), numerous experimental studies aim to substantiate the assumption that infinitesimal doses can elicit biological phenomena similar to those obtained with ponderable doses of the same substances, in order to validate the use of ultra-diluted medications in homeopathic therapy.^31^, ^32^, ^33^

## Conclusion

Even after 230-years since its therapeutic application began, contributing to increased effectiveness in treating various classes of chronic diseases, homeopathy remains marginalized by orthodox scientific knowledge because it is based on principles distinct from conventional medical practice.

Therefore, the dissemination of evidence-based homeopathy before Brazilian medical institutions is a strategy to disseminate existing knowledge and research, strengthening homeopathy as a medical specialty.

In order to clarify for physicians and other health professionals the principles, application method, and scientific evidence of the homeopathic model, the authors discuss in this article the epistemological, experimental, and clinical aspects that guide good homeopathic practice, bringing them minimum subsidies to evaluate any homeopathic treatments to which their patients are submitted, as well as informing them about a therapeutic alternative of great value and application.

Since homeopathy is indicated as an adjuvant treatment for various health disorders and types of diseases, the conduct of the homeopathic physician must follow a specific scheme in order to ensure that the premises and protocols intrinsic to the model are considered, as the quality of the prescription is directly related to the case assessment (global homeopathic semiology), the selection of symptoms (evaluation and repertorization of signs and symptoms), and the differential diagnosis between the various medicinal hypotheses (individualization of the homeopathic medicine) through the study of Homeopathic Materia Medica.

As an integrative and complementary therapeutic proposal, homeopathy can add efficacy, effectiveness, efficiency, and safety to medical practice, acting in a curative and preventive way, reducing symptomatic manifestations and the predisposition to illness, with low cost and minimal adverse events.

## Funding

This research did not receive any specific grant from funding agencies in the public, commercial, or not-for-profit sectors.

## Data availability

The datasets generated and/or analyzed during the current study are available from the corresponding author upon reasonable request.

## Declaration of interest

The authors declare that they have no known competing financial interests or personal relationships that could have appeared to influence the work reported in this paper.
